# ‘I don’t know if there’s a happy ending to this story’: An analysis of prostate cancer narratives in a follow-up setting

**DOI:** 10.1177/13634593251358052

**Published:** 2025-07-28

**Authors:** Laura Lahti, Piia Jallinoja

**Affiliations:** Tampere University, Finland; Tampere University, Finland

**Keywords:** cancer experience, cancer narratives, cancer stories, prostate cancer, storylines

## Abstract

Prostate cancer, the most common cancer among Finnish men, has a high survival rate. Treatment options vary from active surveillance to radical treatments, with potential long-term or permanent side effects. Traditional cancer narratives frame cancer as a tragedy or a hero story and thus fail to capture the chronic nature of prostate cancer and its impacts on patients’ lives. This study analyses the narratives of 22 prostate cancer patients, interviewed twice (1 and 3 years after diagnosis). We found two recurring storylines of prostate cancer narratives, one from radically treated men and the other from men under active surveillance. We analysed how cultural plot types – hero story, tragedy, comedy and irony – appear in the narratives. While tragedy dominated narratives, re-interviews also revealed irony as the tragic elements were caused by the treatment side effects, not the cancer itself. Comedic elements emerged when side effects were reframed as symptoms of ageing. Narratives took on heroic features if the cancer was cured or non-aggressive. The findings underscore the importance of diverse cultural representations to reflect the multifaceted experience of living with prostate cancer and the need for long-term support with the physical and psychological aspects of prostate cancer.

## Introduction

Prostate cancer is the most common cancer among men in Finland (Seppä et al., 2023) and Europe (Dyba et al., 2021) and the second most common cancer among men worldwide (Barsouk et al., 2020). Prostate cancer particularly affects the lives of older men, as the average age of new Finnish prostate cancer patients is 70 years. Despite the high prevalence of prostate cancer, 94% of diagnosed patients in Finland are still alive 5 years after diagnosis (Seppä et al., 2023). Therefore, a large proportion of men survive prostate cancer, making it important to study what life is like when the cancer and the changes it brings are integrated into everyday life.

Depending on the stage of the cancer, prostate cancer can be treated by active surveillance or more radically by prostatectomy, androgen deprivation therapy, radiation therapy or chemotherapy (Barsouk et al., 2020). In Finland, the treatment method is chosen in collaboration between the patient and the doctor, considering the patient’s values and wishes (Prostate cancer: Current Care Guidelines, 2025).

There are some characteristics in the treatment path of Finnish prostate cancer patients that are important to understand at the outset of this paper. The first encounter with the urologist where a man receives his diagnosis involves other important aspects as well. He is informed about the treatment options and is asked by the doctor to make the decision on his treatment (Prostate cancer: Current Care Guidelines, 2025). Consequently, essential information is provided, and decisions are made at a very fast pace. In addition, androgen deprivation therapy may even be started at the same time. Even the cancer diagnosis itself can be paralysing, when it is challenging to absorb all the information about the side effects of the treatments. Additionally, the side effects may seem distant when a man is worried about surviving.

Radical prostate cancer treatments often cause temporary, long-term, or even permanent side effects such as urinary tract dysfunction, changes in sexuality and erectile dysfunction, bowel symptoms and hormonal symptoms (Donovan et al., 2023). These side effects have been found to significantly impact the quality of life of prostate cancer patients (Green, 2024; Hoffman et al., 2017; Lehto et al., 2017). Active surveillance means that prostate cancer is not treated curatively but PSA levels are monitored regularly. It can cause patients anxiety, worry and fear related to aspects such as the spread of cancer, which may lead to choosing a more radical treatment than what is actually needed (Hedestig et al., 2003; James et al., 2022).

As most prostate cancer patients live long after their diagnosis, the prevalence and severity of the treatment side effects are significant concerns for patients (Hamdy et al., 2023; Talvitie, 2024). Thompson and Futterman (2022) found in their review that life after surgical prostate cancer treatment requires strong adapting to body changes and rebuilding masculinity. In this context, masculinity is viewed as a **social construct** – one that can be challenged and reshaped by illness and ageing. The review highlights how prostate cancer and its treatments can disrupt traditional masculine norms, such as physical strength, sexual performance and emotional restraint, prompting men to renegotiate their masculinity in later life (Thompson and Futterman, 2022). Bowie et al. (2022) also suggested that life after prostate cancer is about adaptation, especially to changes in sexuality and masculinity.

According to earlier studies, it is easier to adapt to prostate cancer and the side effects brought about by its treatments at an older age compared to being younger (Seale and Charteris-Black, 2008; Thompson and Futterman, 2022). This is thought to be the case because the regular somatic changes caused by ageing are highly similar to the side effects of both prostate cancer and its treatments (Green, 2024; Jonsson et al., 2010; Korfage et al., 2006; Lahti and Ojala, 2022; Thompson and Langendoerfer, 2016; Vainionpää and Topo, 2005).

Given the above-described realities of prostate cancer and its treatment side effects, the cultural representations of prostate cancer seem to be too narrow, as previously stated. Johnson (2021) suggested that prostate cancer patients have no representations other than those of the age-related old and impotent man. Furthermore, others (Clarke, 2004; Clarke and Everest, 2006; Clarke and van Amerom, 2008; Halpin et al., 2009) have shown that prostate cancer presentation in the mass media typically contain only a medical perspective. However, previous narrative studies have shown that even if the medical perspective is important, prostate cancer has an impact on all areas of life, and experiences of different situations are intertwined in the everyday life of a cancer patient (Corbally et al., 2023; Gray et al., 2005; van der Kamp et al., 2022). Indeed, some have suggested that prostate cancer patients would benefit from diverse, relatable cultural representations in different situations (Green, 2006; Jensen et al., 2010; Mazor et al., 2010).

Available representations and story models play a significant role when the illness is considered in everyday life (Bury, 2001). Surviving chronic cancer is not about beating it but about living with it and accepting it as part of one’s life. This process can be long and requires a lot of adjustment (Broom et al., 2018). Due to the specific characteristics of prostate cancer, the story can continue for a long time with uncertainty while men are in a liminal state (Pietilä et al., 2018). For everyday life to continue, men must integrate the illness and the changes it brings into their lives. Narratives are one previously stated way to adapt to illness (Bury, 2001). A narrative approach helps to understand how prostate cancer becomes part of men’s life. In this article, we examine narratives about life with prostate cancer in a follow-up setting including differently treated men – those who underwent radical treatments, and those under active surveillance.

### Theoretical framework

A narrative approach was chosen as the theoretical framework for this study to deeply understand narrated experiences and storylines of prostate cancer in different phases. The approach has been increasingly used to understand illness and cancer experience (Canella et al., 2023; Ezzy, 2000; Frank, 2013; Gray et al., 2005; Reffner Collins et al., 2024). People create their own cancer narratives to reflect on their illness in relation to cultural expectations and others in similar situation, which helps them cope better (Bury, 2001; Hänninen, 1999). Additionally, the narrative approach is valuable for revealing the variation in narratives vis-à-vis the limited cultural representations available for prostate cancer patients.

Narratives have been analysed following certain plot types first in literature studies (Murray, 1989), and later in social sciences (Alasuutari, 1998; Hänninen, 1999) and research on illness narratives (Bury, 2001). There are four commonly and repeatedly used main plot types in the Western story tradition: hero story, tragedy, comedy and irony. These plot types, grounded in Western narrative traditions, structure life events as storylines. We anticipated that elements of these plot types might be reflected in how men described their experiences. However, it is important to note that these plot types are analytical constructs applied by the researchers, not terms used by the men themselves. For example, the men did not explicitly say, ‘this was a tragedy’, but they used word choices that supported our interpretations of plot types, such as ‘*horrible*’, ‘*sad*’ or ‘*depressing*’. The plot types help to understand and interpret the complex realities of living with prostate cancer over the long term, and provide insights into how patients navigate their illness, manage its psychological and physical challenges, and find meaning in their experiences. The plot types are briefly presented below.

In a hero story, the initial situation is a state of harmony, which is threatened by the forces of evil. The hero manages to overcome the threat with a fight. After struggles, the main character emerges as a hero who has survived conflicts. The main character of a hero story is characterised by strength and moral superiority (Frye and Bloom, 2000; Hänninen, 1999; Murray, 1989).

Tragedy has a heavy and sad undertone. Dark forces seem to destroy the main character, and he or she fails in defeating the struggles. The basic quality of the main character of the tragedy is innocence (Frye and Bloom, 2000; Hänninen, 1999; Murray, 1989).

In a comedy, the starting point is a situation where society is restricting the individual. The contradiction between society and the individual escalates into conflict. With the conflict the rules of normal life expire. The result is a new, healthier social community. The basic features of the main character include having desires, human weaknesses and needs (Frye and Bloom, 2000; Hänninen, 1999; Murray, 1989).

Irony calls the purity and simplicity of all other plot types into question: nothing in it is purely good or bad, and the way other types of plots present things as being one or the other is only a form of structuring experience. Social standards are set in a new light. The main character in irony is characterised by intelligence and a desire to break boundaries (Frye and Bloom, 2000; Hänninen, 1999; Murray, 1989).

When these plot types are used to describe real-life events, the narratives are typically less dramatic compared to literary and fictional stories. For example, comedies are not necessarily funny and the battles heroes go through are not necessarily exciting (Hänninen, 1999).

Public cancer stories typically take the form of a tragedy or a hero story (Bury, 2001; Murray, 1989, 2010). For example, on social media, cancer has been portrayed as a battle and survivorship (Ma et al., 2023). In the print media cancer has been seen as a killer and a threat to human life. The victim can either survive and come out as the winner or die and emerge as the loser (Hanne and Hawken, 2007). In *tragedy*, health is strongly associated with happiness and as a result, getting sick poses a threat not only to health but to overall wellbeing and happiness. If the fight against cancer does not bring good results, the story ends with death or losing control of life. The patient is typically seen as an innocent victim. By contrast, in a *hero story*, surviving cancer patients are presented as fighters and winners (Bury, 2001; Murray, 1989, 2010).

Due to the special chronic nature of prostate cancer described above, these cultural story models of cancer may not stand as realistic representations of prostate cancer patients. In this article, we will show how different plot types are presented in the narratives of prostate cancer. This study deepens the understanding of what life with prostate cancer can be like as time passes between diagnosis and the later stages of living with cancer and its side effects. The examination of narrative elements expands the prevailing medical view of the flow of narratives focusing on diagnosis, treatments, and possible recovery.

This qualitative study analyses interviews of 22 men, covering the period from diagnosis to post-treatment. The study asks what kinds of narratives prostate cancer survivors tell in the interviews, what kind of storylines of narratives are, and how four traditional plot types appear in the narratives.

## Materials and methods

### Data

**Figure 1.**
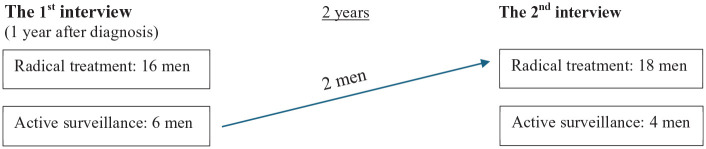
Description of the dataset.

The data of this study consists of individual interviews with 22 prostate cancer patients collected in a follow-up setting between 2019 and 2022 (Figure 1). The interviews were conducted twice with each participant, resulting in a total of 44 interviews. The interviewees were 66–86 years old (an average age of 73) at the time of the first interview. All interviewees were retired. The Finnish pension system enables individuals to live without obligations after leaving paid employment.

The data were collected as part of research project ‘QPro3: Life-satisfaction after prostate cancer treatments: a follow-up study with interview and survey data’ that analyses the quality of life of prostate cancer patients with surveys and individual interviews. The survey data comprised 626 participants diagnosed with prostate cancer. Of these 626 participants, each year, 20 participants were randomly selected by research group for qualitative interviews so that they represented different age groups and prostate cancer treatment types. None of those invited refused to be interviewed. In this article, we analyse interview pairs that were completed (i.e. both interviews were conducted and transcribed) by November 2022 when work on this paper began. The first author was the coordinator of the research project and collected both the survey and interview data with research group.

The selected participants were sent an invitation letter to participate in the interview, followed by an invitation call by a project researcher. None of the invited participants refused to be interviewed. Both in the invitation letter and during the phone call, the participants were provided with information about the research. They also had the opportunity to ask questions. The participants gave written informed consent to participate in the study. Initially, the interviews were conducted face-to-face at the premises of the university, but later by telephone due to the COVID-19 pandemic. The interviews were recorded and transcribed. The project was granted research permission by the Pirkanmaa Hospital District and approval from the Ethics Committee of University Hospital District.

The difference in the number of radically treated interviewees and those under active surveillance can be seen as a limitation. However, this study aimed to present diverse cancer experiences rather than compare treatment methods. The numbers reflect Finnish treatment practices, where radical treatments are more common.

The interviews were carried out as semi-structured thematic interviews with the aim of providing an opportunity for the interviewees to talk freely (Belina, 2023). Although the themes of the interviews were predetermined (e.g. diagnosis, choice of treatment, side effects, quality of life, relationships), the interviewer’s goal was to allow the interviewees to talk in their typical way and about the things that were important to them. During the interviews, the interviewees reported their experiences, feelings, opinions, and expectations related to the themes, which formed a description of the events (Holstein and Gubrium, 1995).

Thematic interviews have been criticised for producing narrative data, as the interviewer might control the conversation too much. Open discussion addresses criticisms of interviews as a data collection method in narrative analysis (e.g. Flick, 2022). While the interviews were conducted using a semi-structured thematic guide, our approach emphasised openness and flexibility. As Maxwell (2013) notes, qualitative interviews can vary in the degree to which they are structured. In our study, the goal was to allow participants to shape the direction and content of the conversation despite predefined themes. The openness enabled the emergence of topics that were not predefined before the interview. This approach aligns with the Maxwell’s (2013) emphasis on understandings participants’ perspectives in their own terms.

Conducting the interviews in a follow-up setting enabled an analysis of a period longer than would have been the case with only one interview of each man. In addition, by the time of the second interview, most of the interviewees had already finished their treatments, which allowed them to provide in-depth descriptions of their lives after the treatments, including possible side effects, and how the cancer journey evolved.

### Analysis

The data were analysed with narrative analysis (Fina and Georgakopoulou, 2015; Herman and Vervaeck, 2019). There are many ways to implement narrative analysis (e.g. Labov, 1973; Lieblich et al., 1998; Polkinghorne, 1995; Riessman, 2008). Broadly speaking, narrative analysis can be divided into an *analysis of narratives*, where the narratives are divided into parts, after which the descriptions related to a certain issue are brought together (Lieblich et al., 1998; Polkinghorne, 1995) and *narrative analysis*, where the goal is to find events and plots that are mentioned in several interviews, and to synthesise the revealed story types (Polkinghorne, 1995). In this article, we employed narrative analysis with the aim of locating typical storylines of prostate cancer narratives.

As soon as we became familiar with the data, we noticed that the interviewees’ ways of talking about their illness had a similar beginning. After the beginning, the narratives diverged into two storylines. We decided to choose a narrative analysis method, in which we constructed example stories from these two storylines. With this method, we were able to show a similar beginning and how and for what reasons the storylines diverge. The method also enabled optimal utilisation of the follow-up setting and the presentation of narratives in their entirety over the long term. Our aim was also to explore how traditional plot types appear in the narratives. The analysis was conducted using the process described below:

In the first phase, the first author read through all the transcribed interviews several times and took initial notes on them. Second, the episodes in the interviews were located and coded according to the stages of the disease (e.g. suspicions of the disease, seeking treatment, choosing the treatment method, feelings about the diagnosis, side effects). Then, the episodes were arranged in chronological order for each man. When re-reading the chronologically organised episodes, we paid attention to the narrative tone, such as positive and negative points, and the main themes of the narratives.

Two repeated storylines of prostate cancer narratives were recognised, one among those who underwent radical treatment and the other among those in active surveillance. These were condensed into two main storylines. We call the storylines the *stories* of Esa and Hermanni, which are the names given by researchers to constructed stories. We use the term story here to denote the researchers’ constructions and interpretations of the events (Culler, 2002). This also distinguishes these two stories from the narratives of each of the 22 interviewees. Esa’s story represents the storyline of radically treated men, while Hermanni’s story represents the storyline of men under active surveillance. In the results section, we present the stories side by side to highlight their similarities and differences.

In the last phase of analysis, we analysed how the traditional plot types, as presented by Frye and Bloom (2000), Murray (1989, 2010) and Bury (2001), appear in the prostate cancer narratives to generate new knowledge about illness narratives. Characteristics of plot types, as defined in previous literature, served as our criteria for interpreting the interviewee’s statements. In this phase, we returned to the interview data and made sure that these plot types could also be found in the interviews. While the presented typification is not necessarily exhaustive, we were interested in the four plot types mentioned above. We illustrate the found plot types mainly with the interview extracts, but also with examples from Esa’s and Hermanni’s stories. Codes were used to denote the interviewees’ treatment method. Men who had undergone radical treatment (surgery/androgen deprivation therapy/radiation therapy/chemotherapy) were coded as RT (RT1, RT2 etc.), and interviewees under active surveillance as AS (AS1, AS2 etc.). Interviewees who were under active surveillance during the first interview but who had transitioned to radical treatment before the re-interview were coded as ASRT (ASRT1, ASRT2).

## Results

We found two main storylines of prostate cancer narratives, one common among those who underwent radical treatment (Esa) and the other among those under active surveillance (Hermanni). The section begins with Esa’s and Hermanni’s prostate cancer stories, which are descriptive stories based on narratives and storylines recurring in the interviews. Following the stories, the occurrence of cultural plot types within the narratives is presented.

**Table:** 

Esa and Hermanni, retired and married men in their seventies, enjoy jogging, theatre and spending time with their grandchildren. For years, they experienced frequent night-time urination, attributing it to age. Elevated PSA levels led to prostate examinations and cancer diagnosis, after which their stories began to move forward in different ways.	
Esa’s cancer was stubborn but not metastasised. The word ‘cancer’ evoked fear and discomfort in him. He believed prostate cancer was inevitable for older men. However, the boldness of the cancer surprised him, lowering his mood. Esa was told about treatment options in a treatment consultation. He chose surgery, believing it was the quickest way to eliminate the cancer. The side effects were not discussed much, or at least Esa did not remember them being discussed. His primary concern was to treat the cancer. The surgery went well. After the surgery, Esa had no urinary retention capacity and had to wear incontinence pads. Esa read online about the factors facilitating urinary incontinence. He controlled his drinking and exercised his pelvic floor muscles, but his condition did not improve. Esa hoped it might get better someday, while also believing that urinary incontinence is common in older men. Esa had to stop exercising due to urinary incontinence. He also gave up cultural events and social activities once enjoyed, wanting to keep his urinary incontinence a secret out of shame. Esa feared the pads would show through his trousers and cause smells. He was uncomfortable changing pads in public restrooms due to the sounds, which he did not want others to hear. Esa lost erectile function after the surgery, which struck at his masculinity and made him feel inadequate for not meeting his spouse’s needs. He received an injection to treat erectile dysfunction and was initially satisfied. However, Esa soon noticed that despite having an erection, he could not have sex because of urinary incontinence. Esa found the urine leakage during sex unpleasant. — *After 2 years —* Esa’s lifestyle was shrinking due to urinary incontinence. He had eliminated most social activities, and his sexual life was non-existent. Esa believed urinary incontinence was a part of later life and that his sexual life should be over at this point. Esa’s mood declined, and he began to regret choosing surgery. When deciding on the treatment, he had been unaware that urinary incontinence would become a lifelong problem. Esa reflected that he might not have chosen the surgery if he had known.	Hermanni’s cancer was small and non-aggressive. He felt strange about the diagnosis but considered it normal since many old men in his village also had prostate cancer. Hermanni believed that as people age, eventually everyone gets prostate cancer. The doctor recommended active surveillance for Hermanni’s non-aggressive prostate cancer. Active surveillance was an unfamiliar concept to Hermanni, but he researched it online. He was surprised to have cancer without immediate treatment but trusted the doctor’s judgement. Hermanni decided to forget about the cancer since he received no treatment and had no symptoms. He managed well, continued his normal life, and the cancer did not bother him. Hermanni had a PSA test every 3 months. The levels remained stable, and no further examinations or treatment changes were needed. Meanwhile, many of Hermanni’s friends underwent radical treatments. One friend also died of prostate cancer. — *After 2 years—* Hermanni became nervous every time the PSA test approached. Although his levels were stable, his anxiety increased. Hermanni’s friends’ experiences made him more aware of his own cancer. His worries about the cancer inside him increased. Thoughts of cancer spreading and dying became more frequent. Hermanni described his situation as a ticking bomb inside him that would eventually explode. He feared the cancer is gradually spreading inside him. Hermanni was worried and scared daily, resulting in a low mood. He did not seek help because he did not know where to go, feeling alone and empty with his worries. Hermanni felt that, for the sake of his peace of mind, he would like to get rid of the cancer, for example by undergoing surgery.

### Plot typification by using cultural plot types

The storyline of the radically treated men showed that cancer was gone, but men were left with urinary incontinence and erectile difficulties as a side effect of the treatment, which significantly restricted their lifestyle. On the other hand, the storyline of the men under active surveillance showed that this treatment method became mentally difficult for men, and they began to worry that there was spreading cancer inside them.

In the following analysis, we apply the analytic concepts of tragedy, comedy, irony and hero story to describe narrative elements in the storylines. The narratives began with the tragedy of falling ill. In both storylines, the men attempted to adapt to the tragedy by altering their perspectives, incorporating comedic elements. Both storylines momentarily transformed into a hero story, either after the illness was cured or by highlighting that the illness was not particularly dangerous. Later, the comedic elements re-emerged as men who underwent radical treatments tried to adapt to the side effects by framing them as changes associated with ageing. Over time, these adaptation strategies became less effective for radically treated men and were ineffective for men under active surveillance. Consequently, the narratives reverted to tragedy. The second interview revealed that, in both storylines, the tragedy stemmed from harms related to the chosen treatment. Irony emerged in the narratives as the treatment and the resulting side effects or anxiety, rather than the cancer itself, became the primary challenge.

As the above short description shows, neither of the storylines fits completely with the plot types of hero story, tragedy, comedy, or irony. Instead, both men who underwent radical treatment and those under active surveillance incorporated elements from each of the four plot types in their approaches to dealing with cancer, its treatments, and rebuilding their lives.

Elements of tragedy dominated both storylines right from the beginning. However, this tragedy slightly differed from the traditionally told cancer tragedy that involves losing the battle against cancer. At first, getting sick was seen as a tragedy, which is a typical plot type when a person’s life is threatened (Bury, 2001; Hanne and Hawken, 2007) as one interviewee also stated: ‘*of course, it* [cancer diagnosis] *felt like the sky was falling. I mean, I was really scared*’ (RT17). The elements of tragedy intensified over time when men realised that the severe side effects of surgery had become permanent and had led to the loss of meaningful things from their lives. This is exemplified by statements such as ‘*I haven’t even thought about going to the theatre or movies in this situation*’ (ASRT1), ‘*hobbies like going to the cottage had to be put on hold*’ (RT15) and ‘*I’m still using those leak guards and there’s some urine leakage. So, sex isn’t really appealing*’ (RT7). For men under active surveillance, anxiety and fear of the cancer spreading grew as time passed, which one interviewee also described: ‘*the hardest part is when you think that there is cancer inside. The thought of cancer keeps spinning in my mind, so sometimes I have to take some tranquilizers to cope. . .*’ (ASRT2).

The narratives show that living with prostate cancer does not necessarily end tragically, but rather potentially continues throughout the rest of the person’s life with elements of tragedy. As one interviewee reflected ‘*I don’t know if there’s a happy ending to this story at all*’ (AS4). Often, elements of tragedy only emerged later, after the radically treated patients have first survived cancer and their focus has shifted to recovery. The final tragedy was caused by the type of treatment and the resulting harm, as one interviewee stated: ‘*even though I’ll survive, it still causes anxiety*’ (AS4).

There are very few heroic elements in the narratives. Regarding the features of a hero story, radically treated men improved from a medical point of view: they survived the cancer like a winner − ‘*the prostate specialist said that it’s beaten now*’ (RT16). For the men under active surveillance, the cancer was initially very mild and not at all dangerous from a medical point of view, such as the following interviewee’s cancer: ‘*It was named as cancer, but it’s the lowest grade, the nice kind of cancer*’ (AS3). In this case, a potentially life-threatening disease can be seen as a threat that the man avoids as a hero. The features of the hero story only emerged in the initial stages of the narratives. When considering life as a whole or quality of life, the side effects of the treatments became prominent. It is noteworthy that the narratives contained elements of the hero story until the end of the radical cancer treatment, but not after that.

We also found elements of comedy in the narratives. In comedy, like in tragedy, something appears to threaten the individual. However, in comedy, issues and threats are seen in a new light due to a change, such as a shift in the person’s thinking or the protagonist viewing things from new perspectives (Frye and Bloom, 2000). Elements of comedy appeared in the narratives of radically treated men with their initial attempts to adapt to prostate cancer and the subsequent interpretations of difficult side effects as changes of ageing and the symptoms, as one interviewee stated ‘*I guess with age it comes that you can limp more often in the toilet and the sex is left out*’ (RT1). In the narratives of men under active surveillance, elements of comedy appeared when they adapted to the cancer diagnosis by shifting their perspective of cancer as a tragic occurrence to one highlighting the prevalence of prostate cancer in men in their age group, like one interviewee also described: ‘*It’s* [prostate cancer] *nothing strange at this age. . .if you take ten men my age, seven have this*’ (AS2). As a result, the men made no efforts to even start a fight with their opponent, cancer or side effects, but instead changed their perspectives and over-adapted to the situation. A significant finding was that over time, framing the situation simply as part of ageing was no longer enough for adapting to side effects. The elements of tragedy began to emerge more strongly in the follow-up interview and superseded the comedy plot type.

Finally, in relation to cultural cancer stories, it is ironic that in the context of prostate cancer the tragedy is caused by the difficult side effects rather than the cancer itself. This means that men are left in a liminal state (Pietilä et al., 2018), and amid uncertainty. Uncertainty leads to reconsidering treatment choices, thinking that perhaps another treatment would have been better for their quality of life. Men under active surveillance contemplated radical treatment as one interviewee also hoped that ‘. . .*they would do something about the cancer, I’ve been like hanging by a thread for years now*’ (AS4). After surgery, men also contemplated their choice, exemplified by an account by one interviewee ‘*well, now I might reconsider after all, maybe I wouldn’t have gone for the surgery at all*’ (RT17). Follow-up interviews challenge traditional cancer narratives by suggesting that the true tragedy lies in the permanent side effects of prostate cancer treatment and the resulting existential uncertainty.

## Discussion

In our study, we found two repeatedly told storylines of prostate cancer: one told by those who received radical treatment and the other by those under active surveillance. The stories of Esa and Hermanni exemplify these narratives and storylines of them.

Radically treated men were cured but faced significant life changes due to side effects. This storyline highlights the physical and psychological challenges associated with radical treatments. The immediate relief of being cured is overshadowed by the long-term side effects, such as urinary incontinence and loss of erectile function, which significantly impact men’s quality of life, leading to social withdrawal, regret, and resignation.

Men under active surveillance lived with the constant worry of untreated cancer. This storyline underscores the psychological burden of living with untreated cancer. The constant monitoring of PSA levels and the threat of cancer spreading contribute to chronic anxiety (also James et al., 2022). The narratives reveal the emotional toll of the ‘watch and wait’ approach, where the absence of physical symptoms is counterbalanced by the psychological distress of living with an ‘internal ticking bomb’.

Both storylines began with the tragedy of diagnosis, attempted adaptation through a comedic reframing of the situation, and returned to tragedy due to treatment consequences. The irony lies in the fact that the elements of tragedy were caused by the treatment side effects, rather than the cancer itself.

Bury’s plot model provided a fruitful structure for the analysis, though we recognise that applying such a framework involves abstraction. The men did not use plot type labels to describe their experiences; rather, we applied these categories as researchers to interpret recurring narrative patterns. As the results showed, these structures occasionally constrained our analysis, as some narratives did not fit neatly into a single plot type depending on the treatment method. The results offer new insights into illness narratives by expanding Bury’s definitions of plot types within the context of prostate cancer. In particular, the concept of tragedy is broadened to include the ongoing uncertainty and the perpetual nature of the cancer journey, which does not always culminate in death. This uncertainty and continuity led men to express regret about their treatment choices and to reflect on whether another treatment might have been better for their quality of life.

However, narratives are not merely reflections of individual cancer experiences. When men narrate their cancer journeys, their stories are influenced by social and cultural factors beyond their personal perceptions. These influences will be discussed in the following sections.

Firstly, men’s pre-cancer experiences, perceptions, and understanding of prostate cancer can shape their narratives (Green, 2006; Mazor et al., 2010). Prostate cancer is common, making it easy for men to compare their situations with others (Lahti et al., 2023). For example, narratives of men under active surveillance included beliefs of friends who had undergone radical treatments and appeared to men under active surveillance to be vibrant and cured. Still, these radically treated men may experience challenging side effects, such as needing to use incontinence pads, losing the ability to have an erection, and finding sex uncomfortable due to urine leakage. Such long-term and difficult side effects of prostate cancer treatments are not necessarily shared among friends.

Secondly, social attitudes towards prostate cancer and the treatment side effects also influence men’s ways of narrating their cancer. The stigma associated with conditions like urinary incontinence and erectile dysfunction (Larkin et al., 2022), along with the lack of open discussions about the emotional impact of living with prostate cancer, can contribute to feelings of isolation and distress (Fanshawe et al., 2023; James et al., 2022). For example, leaking urine during sex is taboo.

Thirdly, the cultural repertoires available for narrating cancer can shape how men talk about their cancer experiences (Green, 2006; Mazor et al., 2010). Traditional cancer narratives often involve a clear-cut battle against the disease, with the patient either tragically succumbing or heroically overcoming it (Canella et al., 2023; Frank, 2013; Hanne and Hawken, 2007; Reffner Collins et al., 2024). Heroic narratives can be empowering, but they can also place pressure on patients who feel they cannot meet these expectations.

Additionally, men draw from other types of cultural representations to help them adapt to the disease and treatment side effects. In both storylines prostate cancer was viewed as even inevitable part of ageing. The representation of an old man, where cancer or side effects is seen as part of the natural ageing process, offers an alternative way to deal with the illness (Bowie et al., 2022; Lahti and Ojala, 2022). It can also provide patients with the opportunity to accept their illness without feeling like failures if they cannot defeat cancer. The representation of an old man can therefore offer patients more space to adapt to the illness and find meaning in their experiences without having to fit into traditional frameworks of cancer narratives. However, our data indicates that this type of adaptation at the beginning of the cancer experience can be detrimental later when side effects start to dominate life too much. The cultural repertoire of prostate cancer and its treatment side effects as normal parts of ageing may turn harmful if it leads men to avoid seeking help for these side effects.

Furthermore, our results showed that psychological distress caused by treatment, or its side effects can lead to treatment regret, which in turn reduces the quality of life (also Fanshawe et al., 2023). Regret is related to higher levels of worry about the cancer spreading, increased awareness of their cancer, fear of death, and concern about treatment side effects (Clark et al., 2001). We agree with Clark et al. (2001) and van Ee et al. (2018) that reduced quality of life may be caused by underlying psychosocial distress and difficulties in communicating with care staff during and years after treatment decision-making. For radically treated men, it was not clear that urinary incontinence could be a lifelong problem and men under active surveillance did not know where to turn for help with their anxiety.

Although the course of cancer and its treatments is not necessarily the same in real life as it is told and experienced, our findings still reflect the current situation in Finland when everything happens fast at the beginning from the perspective of a man diagnosed with prostate cancer. Of course, it is important to get the cancer under control at first, but we also call for patient consideration even after cancer treatments. Broom et al. (2018) also considered similar issues in their study on chronic cancer experiences, noting that the patient’s future and possibly long-term life are underestimated in cancer treatment. Therefore, there is a great need for support in the post-treatment period when long-term challenges arise. While many prostate cancer patients experience good treatment outcomes, our results show that years of living with cancer also entail numerous burdensome issues, often dismissed merely as part of ageing.

Our interviews showed that living with prostate cancer involves dealing with treatment side effects, adapting to lifestyle changes, and managing psychological distress. These narratives highlight ongoing and continuing struggles, uncertainty and adaptations rather than a definitive tragic or heroic outcome, conveying the complexity and nuances of the prostate cancer experience, which cannot be adequately captured by a single narrative archetype.

This study shows that the follow-up time of a qualitative study analysing men’s experiences of prostate cancer should be sufficiently long. We suggest that future studies should examine narratives over an even longer period, repeating interviews every few years. This could result in the tragedy turning into another type of plot or a different kind of tragedy and reveal whether the patients seek help for their side effects, whether their quality of life will improve, and whether the anxiety of those undergoing active surveillance will grow to a point that the patient ends up seeking unnecessarily radical treatment. It could also provide information about how patients under active surveillance who ended up with radical treatments narrate the side effects of the radical treatments.

A cancer diagnosis is often seen as a biographical disruption (Bury, 2001), but based on our results, we can reflect that in the case of prostate cancer, this disruption extends into everyday life as men cope with long-term treatment side effects (Lehto et al., 2017). This ongoing disruption requires men to adapt to new physical and emotional realities, often without a clear endpoint. Narrative approaches to illness emphasise how storytelling helps individuals make sense of chronic conditions (Frank, 2013; Riessman, 2008). In our study, different narrative types served as a tool for managing uncertainty and reframing the prostate cancer experience. Through narrative reconstruction, men were able to find meaning and continuity in their life stories, even as cancer has altered their trajectories (Corbally et al., 2023; van der Kamp et al., 2022). Biographical data, as emphasises by Broom et al. (2018), can further illuminate how men integrate these experiences into their life stories.

Our findings show how prostate cancer narratives evolve over time and how cultural plot types shape the narratives. We argue that prostate cancer should be portrayed more accurately as a chronic condition – often curable but potentially accompanied by long-term or permanent side effects (Bowie et al., 2022; Donovan et al., 2023). This reframing could help men better anticipate the lived realities of treatment and make more informed decisions (Fanshawe et al., 2023). Moreover, improved communication and guidance throughout the illness trajectory, especially post-treatment, could enhance men’s psychological wellbeing and sense of control (Lahti and Ojala, 2022). By highlighting complexity of prostate cancer, our study adds to the growing recognition that chronic illness experiences require more nuanced cultural and clinical representations (Johnson, 2021).

In conclusion, this study contributes to critical health scholarship by showing how men with prostate cancer narrate their experiences not only in relation to the disease, but also to the lasting effects of treatment. By identifying recurring narrative types, we reveal how men make sense of prostate cancer as a chronic, evolving condition. Our findings challenge dominant cure-focused narratives and call for more nuanced representations that reflect the ongoing realities of survivorship.

## References

[bibr1-13634593251358052] Alasuutari P (1998) An Invitation to Social Research. New Delhi: Sage Publications.

[bibr2-13634593251358052] Barsouk A Padala SA Vakiti A , et al. (2020) Epidemiology, staging and management of prostate cancer. Medical sciences 8(3): 28. 32698438 10.3390/medsci8030028 PMC7565452

[bibr3-13634593251358052] Belina A (2023) Semi-structured interviewing as a tool for understanding informal civil society. Voluntary Sector Review 14(2): 331–347.

[bibr4-13634593251358052] Bowie J Brunckhorst O Stewart R , et al. (2022) Body image, self-esteem, and sense of masculinity in patients with prostate cancer: A qualitative meta-synthesis. Journal of Cancer Survivorship 16(1): 95–110. 33963973 10.1007/s11764-021-01007-9 PMC8881246

[bibr5-13634593251358052] Broom A Kenny K Kirby E (2018) On waiting, hauntings and surviving: Chronicling life with cancer through solicited diaries. Sociological Review 66(3): 682–699.

[bibr6-13634593251358052] Bury M (2001) Illness narratives: Fact or fiction? Sociology of Health & Illness 23(3): 263–285.

[bibr7-13634593251358052] Canella C Inderbitzin M Oehler M , et al. (2023) Cancer survival stories: Perception, creation, and potential use case. Health Expectations 26(4): 1551–1561. 37132762 10.1111/hex.13760 PMC10349243

[bibr8-13634593251358052] Clarke J van Amerom G (2008) Mass print media depictions of cancer and heart disease: Community versus individualistic perspectives? Health & Social Care in the Community 16(1): 96–103. 18181819 10.1111/j.1365-2524.2007.00731.x

[bibr9-13634593251358052] Clarke JN (2004) A comparison of breast, testicular and prostate cancer in mass print media (1996–2001). Social Science & Medicine 59(3): 541–551. 15144763 10.1016/j.socscimed.2003.11.018

[bibr10-13634593251358052] Clarke JN Everest MM (2006) Cancer in the mass print media: Fear, uncertainty and the medical model. Social Science & Medicine 62(10): 2591–2600. 16431004 10.1016/j.socscimed.2005.11.021

[bibr11-13634593251358052] Clark JA Wray NP Ashton CM (2001) Living with treatment decisions: Regrets and quality of life among men treated for metastatic prostate cancer. Journal of Clinical Oncology 19(1): 72–80. 11134197 10.1200/JCO.2001.19.1.72

[bibr12-13634593251358052] Corbally M McGarvey C Kestell B (2023) Prostate cancer, radical prostatectomy, recovery, and survivorship: A narrative study of how men make sense of a cancer diagnosis. European Journal of Cancer Care 2023: 1–7.

[bibr13-13634593251358052] Culler J (2002) Story and discourse in the analysis of narrative. In: Culler J (ed.) The Pursuit of Signs: Semiotics, Literature, Deconstruction. Ithaca and New York: Cornell University Press, pp.169–187.

[bibr14-13634593251358052] Donovan JL Hamdy FC Lane JA , et al. (2023) Patient-reported outcomes 12 years after localized prostate cancer treatment. NEJM Evidence 2(4): EVIDoa2300018. 10.1056/EVIDoa2300018 38320051

[bibr15-13634593251358052] Dyba T Randi G Bray F , et al. (2021) The European cancer burden in 2020: Incidence and mortality estimates for 40 countries and 25 major cancers. European Journal of Cancer 157: 308–347. 34560371 10.1016/j.ejca.2021.07.039 PMC8568058

[bibr16-13634593251358052] Ezzy D (2000) Illness narratives: Time, hope and HIV. Social Science & Medicine 50(5): 605–617. 10658842 10.1016/s0277-9536(99)00306-8

[bibr17-13634593251358052] Fanshawe JB Wai-Shun Chan V Asif A , et al. (2023) Decision regret in patients with localised prostate cancer: A systematic review and meta-analysis. European urology oncology 6(5): 456–466. 36870852 10.1016/j.euo.2023.02.005

[bibr18-13634593251358052] Fina AD Georgakopoulou A (2015) The Handbook of Narrative Analysis. Chichester: John Wiley & Sons.

[bibr19-13634593251358052] Flick U (2022) An Introduction to Qualitative Research. London: Sage Publications.

[bibr20-13634593251358052] Frank AW (ed.) (2013) The Wounded Storyteller: Body, Illness, and Ethics, 2nd edn. Chicago, IL: University of Chicago Press.

[bibr21-13634593251358052] Frye N Bloom H (2000) Anatomy of Criticism: Four Essays. Princeton, NJ: Princeton University Press.

[bibr22-13634593251358052] Gray RE Fergus KD Fitch MI (2005) Two black men with prostate cancer: A narrative approach. British Journal of Health Psychology 10(Pt 1): 71–84. 15826335 10.1348/135910704X14429

[bibr23-13634593251358052] Green MC (2006) Narratives and cancer communication. Journal of Communication 56(suppl_1): S163–S183.

[bibr24-13634593251358052] Green R (2024) Experiences and management of urinary incontinence following treatment for prostate cancer: Disrupted embodied practices and adapting to maintain masculinity. Health 28(4): 489–506. 37391939 10.1177/13634593231185266 PMC11151700

[bibr25-13634593251358052] Halpin M Phillips M Oliffe JL (2009) Prostate cancer stories in the Canadian print media: Representations of illness, disease and masculinities. Sociology of Health & Illness 31(2): 155–169. 18983423 10.1111/j.1467-9566.2008.01122.x

[bibr26-13634593251358052] Hamdy FC Donovan JL Lane JA , et al. (2023) Fifteen-year outcomes after monitoring, surgery, or radiotherapy for prostate cancer. New England Journal of Medicine 388(17): 1547–1558. 36912538 10.1056/NEJMoa2214122

[bibr27-13634593251358052] Hanne M Hawken SJ (2007) Metaphors for illness in contemporary media. Medical Humanities 33(2): 93–99. 23674429 10.1136/jmh.2006.000253

[bibr28-13634593251358052] Hänninen V (1999) Inner narrative, life, and change. PhD Thesis, Tampere University, Finland.

[bibr29-13634593251358052] Hedestig O Sandman PO Widmark A (2003) Living with untreated localized prostate cancer: A qualitative analysis of patient narratives. Cancer Nursing 26(1): 55–60. 12556713 10.1097/00002820-200302000-00008

[bibr30-13634593251358052] Herman L Vervaeck B (eds) (2019) Handbook of Narrative Analysis, 2nd edn. Lincoln, NE: University of Nebraska Press.

[bibr31-13634593251358052] Hoffman RM Lo M Clark JA , et al. (2017) Treatment decision regret among long-term survivors of localized prostate cancer: Results from the prostate cancer outcomes study. Journal of Clinical Oncology 35(20): 2306–2314. 28493812 10.1200/JCO.2016.70.6317 PMC5501361

[bibr32-13634593251358052] Holstein JA Gubrium JF (eds) (1995) Active Interview, 1st edn. Thousand Oaks: Sage Publications.

[bibr33-13634593251358052] James C Brunckhorst O Eymech O , et al. (2022) Fear of cancer recurrence and PSA anxiety in patients with prostate cancer: A systematic review. Supportive Care in Cancer 30(7): 5577–5589. 35106656 10.1007/s00520-022-06876-z PMC9135793

[bibr34-13634593251358052] Jensen JD Moriarty CM Hurley RJ , et al. (2010) Making sense of cancer news coverage trends: A comparison of three comprehensive content analyses. Journal of Health Communication 15: 136–151. 20390983 10.1080/10810730903528025

[bibr35-13634593251358052] Johnson E (2021) A Cultural Biography of the Prostate. London: The MIT Press.

[bibr36-13634593251358052] Jonsson A Aus G Berterö C (2010) Living with a prostate cancer diagnosis: A qualitative 2-year follow-up. Ageing Male 13(1): 25–31. 10.3109/13685530903424170 19903116

[bibr37-13634593251358052] Korfage IJ Hak T de Koning HJ , et al. (2006) Patients’ perceptions of the side-effects of prostate cancer treatment—A qualitative interview study. Social Science & Medicine 63(4): 911–919. 16798130 10.1016/j.socscimed.2006.01.027

[bibr38-13634593251358052] Labov W (1973) Language in the Inner City: Studies in the Black English Vernacular. Pennsylvania, PA: University of Pennsylvania Press.

[bibr39-13634593251358052] Lahti L Ojala H (2022) It just belongs to this age” – interpretations of ageing as part of living with prostate cancer. Gerontologia 36(4): 362–374.

[bibr40-13634593251358052] Lahti L Ojala H Pietilä I (2023) Urinary leakage, everyday life, and quality of life. An Interview study on the adaptation of prostate cancer patients to urinary dysfunction. Kuntoutus 46(4): 5–17.

[bibr41-13634593251358052] Larkin D Birtle AJ Bradley L , et al. (2022) A systematic review of disease related stigmatization in patients living with prostate cancer. PLoS One 17(2): e0261557. 10.1371/journal.pone.0261557 PMC8836305 35148315

[bibr42-13634593251358052] Lehto US Tenhola H Taari K , et al. (2017) Patients’ perceptions of the negative effects following different prostate cancer treatments and the impact on psychological well-being: A nationwide survey. British Journal of Cancer 116(7): 864–873. 28222069 10.1038/bjc.2017.30 PMC5379142

[bibr43-13634593251358052] Lieblich A Tuval-Mashiach R Zilber T (1998) Narrative Research: Reading, Analysis, and Interpretation. Thousand Oaks, CA: Sage Publications.

[bibr44-13634593251358052] Maxwell JA (2013) Qualitative Research Design: An Interactive Approach. Thousand Oaks: Sage Publications.

[bibr45-13634593251358052] Ma Z Ma R Zhao X , et al. (2023) Stories that engage the audience: An investigation of popular breast cancer narratives on social media. Telematics and Informatics 85: 102048.

[bibr46-13634593251358052] Mazor KM Calvi J Cowan R , et al. (2010) Media messages about cancer: What do people understand? Journal of Health Communication 15(Suppl 2): 126–145. 20845199 10.1080/10810730.2010.499983 PMC2947749

[bibr47-13634593251358052] Murray K (1989) The construction of identity in the narratives of romance and comedy. In: Gergen KJ Shotter J (eds) Texts of Identity. London: Sage Publications, pp.176–193.

[bibr48-13634593251358052] Murray K (2010) The construction of identity in the narratives of romance and comedy. Available at: https://kevinmurray.com.au/text/the-construction-of-identity-in-the-narratives-of-romance-and-comedy (accessed 4 December 2024).

[bibr49-13634593251358052] Pietilä I Jurva R Ojala H , et al. (2018) Seeking certainty through narrative closure: Men’s stories of prostate cancer treatments in a state of liminality. Sociology of Health & Illness 40(4): 639–653. 29430679 10.1111/1467-9566.12671

[bibr50-13634593251358052] Polkinghorne DE (1995) Narrative configuration in qualitative analysis. International Journal of Qualitative Studies in Education 8(1): 5–23.

[bibr51-13634593251358052] Prostate cancer: Current Care Guidelines (2025) Working group set up by the Finnish Medical Society Duodecim and the Finnish Urologist Society. Helsinki: The Finnish Medical Society Duodecim. Available at: https://www.kaypahoito.fi/hoi11060#K1 (accessed 12 May 2025).

[bibr52-13634593251358052] Reffner Collins MK Lazard AJ Hedrick McKenzie AM , et al. (2024) “It’s nothing like cancer”: Young adults with cancer reflect on memorable entertainment narratives. Health Communication 39(3): 552–562. 36746916 10.1080/10410236.2023.2174403

[bibr53-13634593251358052] Riessman CK (2008) Narrative Methods for the Human Sciences. London: Sage Publications.

[bibr54-13634593251358052] Seale C Charteris-Black J (2008) The interaction of age and gender in illness narratives. Ageing and Society 28(7): 1025–1045.

[bibr55-13634593251358052] Seppä K Tanskanen T Heikkinen S , et al. (2023) Cancer 2021. Statistical Report on the Cancer Situation in Finland. Helsinki: Finnish Cancer Registry.

[bibr56-13634593251358052] Talvitie AM (2024) Quality of life after prostate cancer treatment: A mixed methods study exploring patients’ experiences of quality of life and its measurement. PhD Thesis, Tampere University, Finland.

[bibr57-13634593251358052] Thompson EH Futterman AM (2022) Body talk and resilience: Aging men’s experiences with mastectomy and prostatectomy. Journal of Aging Studies 61: 21. 10.1016/j.jaging.2022.101010 35654545

[bibr58-13634593251358052] Thompson EH Langendoerfer KB (2016) Older men’s blueprint for “being a man.” Men and Masculinities 19(2): 119–147.

[bibr59-13634593251358052] Vainionpää KJ Topo P (2005) The making of an ageing disease: The representation of the male menopause in Finnish medical literature. Ageing and Society 25(6): 841–861.

[bibr60-13634593251358052] van der Kamp J Betten AW Krabbenborg L (2022) In their own words: A narrative analysis of illness memoirs written by men with prostate cancer. Sociology of Health & Illness 44(1): 236–252. 34855224 10.1111/1467-9566.13412 PMC9300072

[bibr61-13634593251358052] van Ee IB Hagedoorn M Smits CHM , et al. (2018) This is an older men’s world: A qualitative study of men’s experiences with prostate cancer. European Journal of Oncology Nursing 37: 56–64. 30473052 10.1016/j.ejon.2018.11.002

